# *FAM20A*: a potential diagnostic biomarker for lung squamous cell carcinoma

**DOI:** 10.3389/fimmu.2024.1424197

**Published:** 2024-06-25

**Authors:** Yalin Zhang, Qin Sun, Yangbo Liang, Xian Yang, Hailian Wang, Siyuan Song, Yi Wang, Yong Feng

**Affiliations:** ^1^ Center for Critical Care Medicine, Sichuan Academy of Medical Science and Sichuan Provincial People’s Hospital, University of Electronic Science and Technology of China, Chengdu, Sichuan, China; ^2^ Clinical Immunology Translational Medicine Key Laboratory of Sichuan Province, Center of Organ Transplantation, Sichuan Academy of Medical Science and Sichuan Provincial People’s Hospital, Chengdu, Sichuan, China; ^3^ Department of Neuroscience, Baylor College of Medicine, Houston, TX, United States; ^4^ Department of Otorhinolaryngology Head and Neck Surgery, Sichuan Provincial People’s Hospital, University of Electronic Science and Technology of China, Chengdu, China

**Keywords:** LUSC, Fam20A, tumors, immunotherapy, DNA repair, radiotherapy

## Abstract

**Background:**

Lung squamous cell carcinoma (LUSC) ranks among the carcinomas with the highest incidence and dismal survival rates, suffering from a lack of effective therapeutic strategies. Consequently, biomarkers facilitating early diagnosis of LUSC could significantly enhance patient survival. This study aims to identify novel biomarkers for LUSC.

**Methods:**

Utilizing the TCGA, GTEx, and CGGA databases, we focused on the gene encoding Family with Sequence Similarity 20, Member A (*FAM20A*) across various cancers. We then corroborated these bioinformatic predictions with clinical samples. A range of analytical tools, including Kaplan-Meier, MethSurv database, Wilcoxon rank-sum, Kruskal-Wallis tests, Gene Set Enrichment Analysis, and TIMER database, were employed to assess the diagnostic and prognostic value of *FAM20A* in LUSC. These tools also helped evaluate immune cell infiltration, immune checkpoint genes, DNA repair-related genes, DNA methylation, and tumor-related pathways.

**Results:**

*FAM20A* expression was found to be significantly reduced in LUSC, correlating with lower survival rates. It exhibited a negative correlation with key proteins in DNA repair signaling pathways, potentially contributing to LUSC’s radiotherapy resistance. Additionally, *FAM20A* showed a positive correlation with immune checkpoints like CTLA-4, indicating potential heightened sensitivity to immunotherapies targeting these checkpoints.

**Conclusion:**

*FAM20A* emerges as a promising diagnostic and prognostic biomarker for LUSC, offering potential clinical applications.

## Introduction

Lung squamous cell carcinoma (LUSC), comprising about 20% of lung carcinomas, is a significant public health concern (1). Despite advancements in surgical techniques and the introduction of innovative treatments like chemotherapy and gene-specific immunotherapies targeting immune checkpoints, therapeutic results remain suboptimal. Notably, the treatment of LUSC presents a formidable challenge in lung cancer management, primarily because diagnoses often occur at advanced cancer stages (2). Consequently, the discovery of novel biomarkers for early diagnosis and predicting therapeutic outcomes is of paramount importance for patients with LUSC.

The Family with Sequence Similarity 20 (*FAM20*) gene family encompasses three members: *FAM20A*, *FAM20B*, and *FAM20*C (3). These genes encode kinases known for phosphorylating secreted proteins and proteoglycans (4). Despite their origin from the same spliceosomes, each of these proteins exhibits unique functions across various tissue (5). *FAM20C*, for instance, is ubiquitously expressed across all tissues and is particularly notable in cancers such as breast cancer (6). Our prior research has identified *FAM20C* as an oncogene in glioblastoma (7). Unlike FAM20C, FAM20A is predominantly expressed in lactating mammary glands and dental matrices (8), and has been shown to form a functional heterotetrameric complex with FAM20A, enhancing its enzymatic activity (9). As a pseudokinase without kinase activity, FAM20A does not facilitate phosphoryl transfer and serves as a paralog of FAM20C (10). Consequently, *FAM20A* has garnered less research focus, though some studies have linked its variants to amelogenesis imperfecta (AI), nephrocalcinosis (NC), and ectopic calcification (EC) (11), highlighting its critical role in several diseases. Bioinformatic analyses have indicated *FAM20A*’s upregulation in papillary thyroid carcinoma, associated with immune infiltration (12), and its potential to predict early lymphatic metastasis in thyroid cancer (13). Whole-genome sequencing studies have also correlated *FAM20A* mutations with parathyroid carcinoma (14), suggesting its implication in the pathogenesis of thyroid and parathyroid cancers. However, the role of *FAM20A* in LUSC remains elusive. In this study, utilizing the TCGA database, we explored *FAM20A* in LUSC and uncovered its tumor-suppressive function.

Initially, our analysis of the FAM family revealed that *FAM20A* potentially assumes a tumor-suppressive role in LUSC development, a hypothesis we subsequently confirmed with clinical samples. We then juxtaposed *FAM20A* with *FAM20C* to evaluate the influence of *FAM20A* on patient outcomes, focusing on overall survival, disease-specific survival, and progression-free survival. Further, in pursuit of a clinically relevant approach for LUSC treatment, we investigated the relationship between *FAM20A* and DNA repair signaling pathways related to radiotherapy, as well as its association with immune checkpoints and immune cell infiltration in the context of immunotherapy. Our research marks the inaugural study to highlight the significance of *FAM20A* as both a diagnostic and prognostic biomarker for LUSC.

Although the molecular markers and signaling pathways of LUSC have been extensively explored in previous studies, little is known about the functional role of FAM20A in the disease and its underlying mechanisms. Therefore, our study not only explores the significance of FAM20A as a potential diagnostic and prognostic biomarker for LUSC, but also aims to gain insight into its biological function during tumorigenesis and progression. Specifically, we focused on investigating the expression pattern of FAM20A in LUSC tissues and explored its correlation with clinicopathologic features, patient survival, and treatment response. In addition, we explored the molecular pathways and biological processes that FAM20A may be involved in, such as DNA repair, cell proliferation, and immune regulation, through a combination of bioinformatics analysis and clinical sample validation. This research allows us to gain a deeper understanding of the functional mechanisms of FAM20A in LUSC and providing a theoretical basis for future therapeutic strategies.

## Materials and methods

### Single-cell sequencing data

Single-cell sequencing data of LUSC patients was sourced from the ArrayExpress database, specifically from the datasets E-MTAB-6149 and E-MTAB-6653, which include five tumor core samples and five normal tissue samples. This study builds upon findings reported in https://www.nature.com/articles/s41591–018-0096–5. The raw data was uploaded for processing on the 10X Genomics Cloud Analysis platform (https://www.10xgenomics.com/cn/products/cloud-analysis). During the analysis, the “GEM-X Single Cell 3’ Gene Expression” option was selected for the Library or Feature Type, and the Cell Ranger Multi v8.0.0 pipeline was employed. The transcriptome reference used was Human (GRCh38) 2024-A. Data analysis was conducted using the SCANPY software. Initial quality control measures filtered out cells based on the percentage of mitochondrial reads and total mapped transcripts. Cells exhibiting more than 10% mitochondrial gene transcripts or fewer than 200 unique molecular identifiers (UMIs)—likely indicative of empty droplets—were excluded. For each treatment group, the 99th percentile of total UMI counts per cell was calculated to determine a threshold for droplet exclusion. Following the initial quality control, data from both groups were combined through data integration techniques to identify shared cell populations across conditions. UMI counts for each dataset were normalized and converted into Pearson residuals using regularized negative binomial regression, which adjusted for variations in sequencing depth across cells. Where necessary, the selection of highly variable genes was conducted by identifying the top 2,000 features from each dataset, ranked by their overall variability.

### Integration, dimensionality reduction and clustering

To consolidate datasets from two distinct tissue groups for unsupervised clustering, we employed the Harmony integration algorithm. This step was followed by Principal Component Analysis (PCA) to reduce dimensionality, capturing gene expression variance across single cells within the integrated dataset. We utilized 50 PCA components and considered 30 nearest neighbors during this process. Cells were subsequently clustered using a graph-based method within the PCA-reduced space. For enhanced visualization of the clusters, non-linear dimensionality reduction was executed using UMAP.

### Cell type annotation

To annotate cell types within clusters, we identified positive cluster markers using the Wilcoxon rank sum test. This analysis was conducted on genes exhibiting a detection rate greater than 10% in any cluster, comparing the target cluster against all other cells. The criteria for significance included a p-value threshold of less than 1e-5 and an average log2 fold change exceeding 0.5. Specific markers were utilized for identification: *Claudin 18 (CLDN18)* for alveolar cells, *CD79A* for B cells, *Claudin 5 (CLDN5)* for endothelial cells, *Calcyphosine (CAPS)* for epithelial cells, *Collagen Type I Alpha 1 Chain (COL1A1)* for fibroblasts, *Lysozyme (LYZ)* for myeloid cells, *CD3D* for T cells, and *Epithelial Cell Adhesion Molecule (EPCAM)* for tumor cells. This approach facilitated the precise delineation of different clusters based on their cellular characteristics.

### Data acquisition

All bioinformatic data were obtained from The Cancer Genome Atlas (TCGA) (https://portal.gdc.cancer.gov/), the Genotype-Tissue Expression (GTEx) database (https://www.gtexportal.org/home/index.html), and the Chinese Glioma Genome Atlas (CGGA) database (http://www.cgga.org.cn) (15, 16). Statistical analyses were conducted using R software following log2 transformation, with visualizations created via the “ggplot2” package.

### Human lung samples

Eighteen LUSC lung tissue samples were obtained from Sichuan Provincial People’s Hospital. Tissues were either immersed in 4% paraformaldehyde (PFA) or stored at -80°C. The study was approved by the Clinical Research Ethics Committee of Sichuan Provincial People’s Hospital (Approval No. 2021–318), with informed consent obtained from all participants.

### Quantitative reverse transcription polymerase chain reaction

This procedure was performed as previously described (7). Primer sequences were listed below: *FAM20C* forward primer: 5’-CCTTCCAGAATTACGGGCAAG-3’; *FAM20C* reverse primer: TGCCTCTCGTAGTCAGAGAAAT; FAM20A forward primer: 5’-AGAGCAGATGAACCTTACCTCC-3’; FAM20A reverse primer: 5’-ATGGCGGTTAATACCCAGGTG-3’; *GAPDH* forward primer: 5’-ACAGCCTCAAGATCATCAGC-3’; *GAPDH* reverse primer: 5’-GGTCATGAGTCCTTCCACGAT-3’.

### Western blotting

The lung tissue was lysed using Lysis buffer (RIPA, Beyotime, China). Protein was segregated by SDS-PAGE and transmitted onto the PVDF membrane (Sigma-Aldrich, USA). After blocking with 5% BSA, the membrane was incubated with polyclonal anti-FAM20A (1:1000, Cat. No. 25258–1-AP, Proteintech, USA), and FAM20C antibody (1:1,000, Cat. No. 25395–1-AP, Proteintech, USA) in a primary antibody dilution buffer (Epizyme, China). Following washing with TBST, the membranes were probed with an HRP-conjugated secondary antibody (1:5,000, Huabio, China). The signal was detected using an ECL substrate (Pierce, USA). Then, the membrane was restored by a stripping buffer (Abcam, USA), blocked, and incubated with internal control as β-actin antibody.

### Immunohistology staining

The protocol, as outlined in our prior publication (7), was employed. The lung specimens were fixed in 4% PFA for subsequent paraffin embedding. Subsequently, each sample was sectioned at a thickness of 5μm. The sections were incubated with FAM20A (1:50, Cat. No. 25258–1-AP, Proteintech, USA) antibody overnight at 4°C.

### Survival analysis

The overall survival (OS), disease-specific survival (DSS), and progress-free interval (PFI) curves for FAM20A and FAM20C in the CGGA database were analyzed using the “Survival Plot” and “Survival Analysis” modules in Gene Expression Profiling Interactive Analysis (GEPIA2) (http://gepia.cancer-pku.cn/) (17, 18).

### Nomogram and calibration

To enhance the prediction of individual patient prognosis in LUSC, we developed a comprehensive prognostic nomogram. This tool facilitates the estimation of overall survival (OS), disease-specific survival (DSS), and progression-free interval (PFI). To ensure its reliability, we generated calibration curves using the ‘rms’ package. These curves serve as a visual validation of the nomogram’s predictive accuracy, effectively aligning the predicted outcomes with actual clinical results.

### Correlation analysis between *FAM20A*, immune cell infiltration, and immune cell markers in LUSC

The ssGSEA algorithm in the “GSVA” (v1.34.0) R package was utilized to assess the tumor infiltration status of 24 immune cell types (19).

### Analysis of DNA methylation status in the CpG islands of the *FAM20A*

MethSurv database (https://biit.cs.ut.ee/methsurv/) was performed to analyze the DNA methylation status in the *FAM20A* gene (20).

### Gene set enrichment analysis

The calculation method that analyzes the statistical significance of priori-defined gene sets and the consistent differences between two biological states is called Gene Set Enrichment Analysis (GSEA) (21). In this study, GSEA was used to create an initial list of gene categories based on the correlation between genes and *FAM20A* expression.

### Statistical analysis

Laboratory experiments were replicated thrice, with data presented as mean ± standard deviation. Student’s t-test and one-way ANOVA were used for group comparisons, considering *P <*0.05 as statistically significant.

### Selection of database and analysis tools

We prioritized databases based on data types, volume, and quality for accurate and comprehensive information. When selecting tools, we focused on functionality and applicability. We used SCANPY for single-cell data analysis, known for its efficiency and accuracy. For survival and gene set enrichment analysis, we opted for GEPIA2 and GSEA, respected for their reliability and utility. Our choices ensure reproducible and interpretable methods, enhancing the credibility of our findings.

## Results

### Single cell sequencing analysis reveals that *FAM20A* is a potential tumor-suppressor gene in LUSC

**Figure 1:**
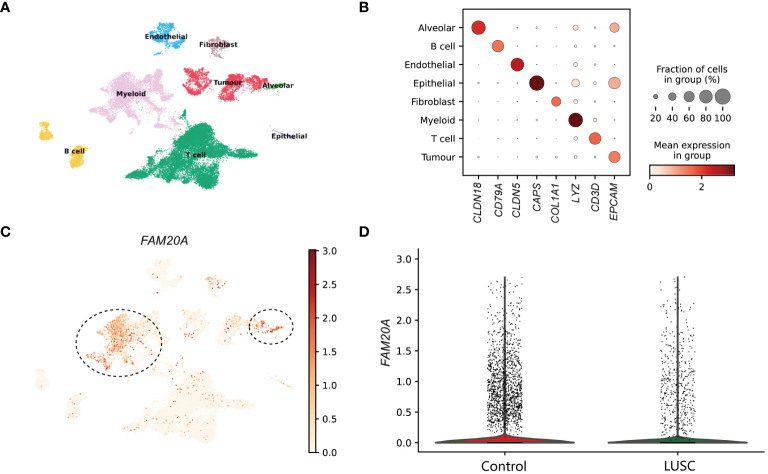
Dissection of *FAM20A* Expression Across Cell Clusters in Lung Squamous Cell Carcinoma. **(A)** UMAP visualization showcasing eight distinct cell clusters derived from single-cell sequencing data of lung tissue samples. **(B)** Dot plot matrix representing the specific marker gene expressions used to identify each cell cluster. The size of the dot indicates the fraction of cells within a cluster expressing the gene, while the color intensity reflects the mean expression level in that cluster. **(C)** Spatial distribution of *FAM20A* gene expression across the integrated cell clusters. Predominant expression in the alveolar cluster is indicated (circled). **(D)** Violin plots illustrating the expression levels of *FAM20A* in control lung tissues versus LUSC tissues. LUSC, Lung squamous cell carcinoma; FAM20A, Family with Sequence Similarity 20, Member A; CLDN18, Claudin 18; CD79A, CD79a Molecule; CLDN5, Claudin 5; CAPS, Calcyphosine; COL1A1, Collagen Type I Alpha 1 Chain; LYZ, Lysozyme; CD3D, CD3d Molecule; EPCAM, Epithelial Cell Adhesion Molecule.

In the examination of LUSC patient data for a detailed analysis, an equitable distribution of samples was employed—consisting of an equal split between control lung tissues and cancer core samples. The application of dimensionality reduction techniques brought to light eight distinctive cellular clusters within the single-cell sequencing data. These clusters were classified as endothelial, fibroblast, alveolar, myeloid, tumor, T cell, B cell, and epithelial (**Figure 1A**), each demarcated by specific marker gene expressions. The precise identification of these clusters was graphically represented in the dot plot of **Figure 1B**, which served as a visual confirmation of the marker gene distribution and prevalence across the identified clusters. A focal point of our findings was the expression pattern of *FAM20A*, a gene of interest due to its suggested role in tumor suppression. Notably, *FAM20A* expression was primarily localized to the alveolar and myeloid cluster, as revealed in the spatial gene expression mapping (**Figure 1C**). The comparative analysis, elegantly depicted in the violin plot of **Figure 1D**, quantitatively highlighted the disparity in *FAM20A* expression between control and LUSC tissues. In the control group, *FAM20A* showed robust expression levels, whereas in LUSC tissues, there was a conspicuous downturn in its expression.

### FAM20A is downregulated in LUSC

**Figure 2:**
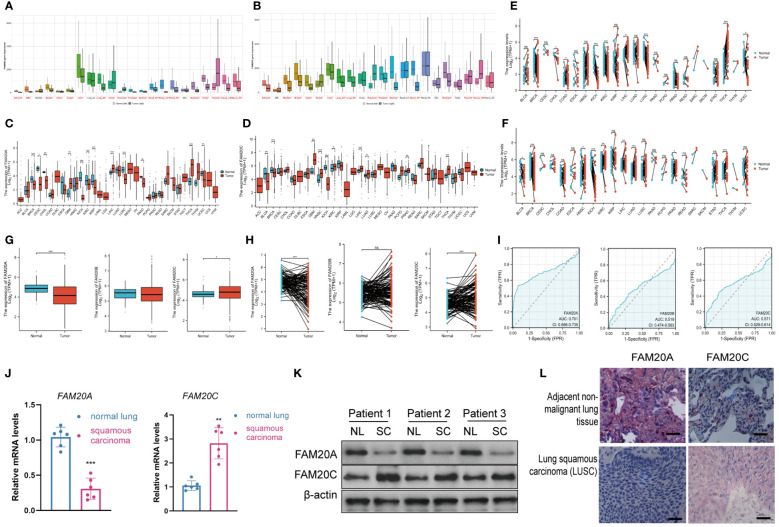
Expression Levels of FAM20A and FAM20C in LUSC. **(A)** *FAM20A* expression across various cancers, as sourced from the GTEx database. **(B)** *FAM20C* expression across different cancers, derived from the GTEx database. **(C)** *FAM20A* expression in 33 cancer types. **(D)** *FAM20C* expression in 33 cancer types. **(E)** Comparison of *FAM20A* expression in tumor versus normal samples. **(F)** Differential expression of *FAM20C* in tumor samples. **(G)** Box plots comparing *FAM20A*, *FAM20B*, and *FAM20C* expression in patients with and without LUSC. **(H)** Expression differences of *FAM20A*, *FAM20B*, and *FAM20C* between LUSC samples and matched non-cancerous samples. **(I)** ROC curve analysis of *FAM20A*, *FAM20B*, and *FAM20C* in normal versus LUSC samples from the TCGA database. **(J)** qRT-PCR analysis for *FAM20A* and *FAM20C*. **(K)** Western blot results for FAM20A and FAM20C. **(L)** Immunohistochemistry (IHC) analysis of FAM20A and FAM20C. Significance levels indicated as *P<0.05, **P<0.01, ***P<0.001. LUSC, Lung squamous cell carcinoma; Fam20A, Family with Sequence Similarity 20, Member A; FAM20B, Family with Sequence Similarity 20, Member B; FAM20C, Family with Sequence Similarity 20, Member C; ACC, Adrenocortical carcinoma; BLCA, Bladder urothelial carcinoma; BRCA, Breast invasive carcinoma; CESC, Cervical squamous cell carcinoma and endocervical adenocarcinoma; CHOL, Cholangiocarcinoma; COAD, Colon adenocarcinoma; DLBC, Lymphoid neoplasm diffuse large B-cell lymphoma; ESCA, Esophageal carcinoma; GBM, Glioblastoma multiforme; HNSC, Head and neck squamous cell carcinoma; KICH, Kidney chromophobe; KIRC, Kidney renal clear cell carcinoma; KIRP, Kidney renal papillary cell carcinoma; LAML, Acute myeloid leukemia; LGG, Brain lower grade glioma; LIHC, Liver hepatocellular carcinoma; LUAD, Lung adenocarcinoma; MESO, Mesothelioma; OV, Ovarian serous cystadenocarcinoma; PAAD, Pancreatic adenocarcinoma; PCPG, Pheochromocytoma and paraganglioma; PRAD, Prostate adenocarcinoma; READ, Rectum adenocarcinoma; SARC, Sarcoma; SKCM, Skin cutaneous melanoma; STAD, Stomach adenocarcinoma; TGCT, Testicular germ cell tumors; THCA, Thyroid carcinoma; THYM, Thymoma; UCEC, Uterine corpus endometrial carcinoma; UCS, Uterine carcinosarcoma; UVM, Uveal melanoma. ns: not significant difference.

To ascertain the roles of *FAM20A* and *FAM20C*, we conducted analyses using public databases. According to the GTEx database, *FAM20A* is downregulated in various cancers (**Figure 2A**). Notably, *FAM20A*’s expression remains stable in lung atypical carcinoid (LUAC), yet it is markedly reduced in LUSC. Concurrently, transcription levels of *FAM20C* are substantially decreased in both LUAC and LUSC (**Figure 2B**). Further validation of *FAM20A* and *FAM20C* expression in tumor samples was performed through mRNA level analysis using the TCGA database. **Table 1** lists the abbreviations and full names of the 33 cancer types included in this study. Results indicate that *FAM20A* expression is diminished in Breast invasive carcinoma (BRCA), Cervical squamous cell carcinoma and endocervical adenocarcinoma (CESC), Cholangiocarcinoma (CHOL), Kidney chromophobe (KICH), Kidney renal clear cell carcinoma (KIRC), Kidney renal papillary cell carcinoma (KIRP), LUSC, Pancreatic adenocarcinoma (PAAD), and Uterine corpus endometrial carcinoma (UCEC), yet elevated in Glioblastoma multiforme (GBM), Liver hepatocellular carcinoma (LIHC), and Thyroid carcinoma (THCA) (**Figures 2C, E**). Conversely, *FAM20C* shows decreased expression in BLCA, BRCA, and KICH, but increased expression in GBM, Head and neck squamous cell carcinoma (HNSC), KIRC, LIHC, Lung adenocarcinoma (LUAD), Prostate adenocarcinoma (PRAD), and THCA (**Figures 2D, F**). Furthermore, LUSC samples demonstrated a notable downregulation of *FAM20A* compared to matched adjacent samples, with no significant change in *FAM20B* expression. In contrast, *FAM20C* expression was upregulated (**Figure 2H**). Analysis of the TCGA database revealed that *FAM20A* expression is significantly downregulated in LUSC patients (*P*<0.001), while *FAM20C* expression is upregulated (*P*<0.05) (**Figure 2G**). *FAM20B* expression did not exhibit significant alterations in LUSC patients (*P*>0.05) (**Figure 2G**). Additionally, we generated ROC curves to evaluate the diagnostic value of *FAM20A*, *FAM20B*, and *FAM20C* in distinguishing LUSC samples from normal ones, using TCGA data. *FAM20A* displayed a high diagnostic potential (AUC=0.7701, CI = 0.666–0.735), whereas *FAM20B* (AUC=0.519, CI = 0.474–0.563) and *FAM20C* (AUC=0.571, CI = 0.529–0.614) exhibited poor diagnostic potential (**Figure 2I**). The transcription and translation levels of *FAM20A* and *FAM20C* were assessed through qPCR, western blot, and immunohistochemistry staining in both normal and LUSC lung tissues. The findings revealed (**Figures 2J-L**) a decrease in *FAM20A* levels and an increase in *FAM20C* levels in LUSC compared to normal lung tissues.

**Table 1:** Abbreviations and details of the 33 cancer types used in this study.

Abbreviation	Detail
Adenoid cystic carcinoma	ACC
breast cancer	BRCA
cervical squamous cell carcinoma and endocervical adenocarcinoma	CESC
cholangiocarcinoma	CHOL
higher expression in glioblastoma multiforme	GBM
head and neck squamous cell carcinoma	HNSC
kidney chromophobe	KICH
Kidney renal clear cell carcinoma	KIRC
Kidney renal papillary cell carcinoma	KIRP
liver hepatocellular carcinoma	LIHC
lung adenocarcinoma	LUAD
lung squamous carcinoma	LUSC
pancreatic adenocarcinoma	PAAD
prostate adenocarcinoma	PRAD
thyroid carcinoma	THCA
uterine corpus endometrial carcinoma	UCEC
Colon adenocarcinoma	COAD
Lymphoid Neoplasm Diffuse Large B-cell Lymphoma	DLBC
Esophageal carcinoma	ESCA
Acute Myeloid Leukemia	LAML
Brain Lower Grade Glioma	LGG
Mesothelioma	MESO
Ovarian serous cystadenocarcinoma	OV
Pheochromocytoma and Paraganglioma	PCPG
Rectum adenocarcinoma	READ
Sarcoma	SARC
Skin Cutaneous Melanoma	SKCM
Stomach adenocarcinoma	STAD
Testicular Germ Cell Tumors	TGCT
Thymoma	THYM
Uterine Carcinosarcoma	UCS
Uveal Melanoma	UVM
bladder urothelial carcinoma	BLCA

### *FAM20A* is associated with LUSC staging

**Figure 3:**
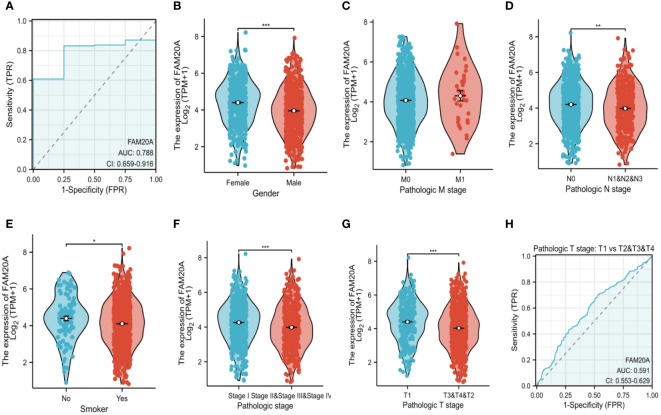
Survival Analysis in LUSC Based on FAM20A and FAM20C. Utilizing the GEPIA2 tool, overall survival (OS), disease-specific survival (DSS), and progression-free interval (PFI) in LUSC were analyzed in relation to the expression of FAM20A and FAM20C, with survival plots provided. Significance levels indicated as *P<0.05, **P<0.01, ***P<0.001. LUSC, Lung squamous cell carcinoma; FAM20A, Family with Sequence Similarity 20, Member A; FAM20C, Family with Sequence Similarity 20, Member C; GEPIA2, Gene Expression Profiling Interactive Analysis 2; OS, Overall survival; DSS, Disease-specific survival; PFI, Progression-free interval.

While *FAM20A* and *FAM20C* do not show a correlation with prognosis, we explored *FAM20A* ‘s association with LUSC stages. Data from 1,041 LUSC patients were extracted from the CGGA database (referenced in **Table 2**). Initially, we assessed *FAM20A*’s sensitivity in LUSC, finding a confidence interval (CI) between 0.659 and 0.916 (**Figure 3A**). We then examined *FAM20A*’s correlation with gender (**Figure 3B**), M stage (**Figure 3C**), N stage (**Figure 3D**), smoking status (**Figure 3E**), and pathological stages (I, II, III, and IV) of the disease (**Figures 3F-H**). Our findings indicate a significant increase in the *FAM20A* score with higher T stage (*P*<0.001), increased lymph node positivity (*P*<0.01), and poorer pathological staging (*P*<0.001) (**Figures 3D, F, G**), with no notable change in advanced metastatic stages (**Figure 3C**). Female patients (*P*<0.001) and smokers (*P*<0.05) exhibited higher *FAM20A* scores (**Figures 3B, E**). In tissue comparisons across different T stages, the AUC was 0.591 (95%CI: 0.553–0.629) (**Figure 3H**).

**Table 2:** Clinical characteristics of the LUSC patients.

Characteristics	Low expression of FAM20A	High expression of FAM20A	P value
n	520	521	
Pathologic T stage, n (%)			< 0.001
T1	120 (11.6%)	170 (16.4%)	
T2	296 (28.5%)	290 (27.9%)	
T3	77 (7.4%)	43 (4.1%)	
T4	26 (2.5%)	16 (1.5%)	
Pathologic N stage, n (%)			0.088
N0	320 (31.4%)	350 (34.3%)	
N1	131 (12.9%)	97 (9.5%)	
N2	58 (5.7%)	56 (5.5%)	
N3	4 (0.4%)	3 (0.3%)	
Pathologic M stage, n (%)			0.737
M0	412 (50.9%)	365 (45.1%)	
M1	16 (2%)	16 (2%)	
Pathologic stage, n (%)			< 0.001
Stage I	240 (23.3%)	301 (29.3%)	
Stage II	161 (15.6%)	126 (12.2%)	
Stage III	99 (9.6%)	69 (6.7%)	
Stage IV	16 (1.6%)	17 (1.7%)	
Primary therapy outcome, n (%)			0.195
CR	307 (37.9%)	335 (41.4%)	
PR	7 (0.9%)	4 (0.5%)	
PD	53 (6.5%)	49 (6%)	
SD	20 (2.5%)	35 (4.3%)	
Gender, n (%)			< 0.001
Female	167 (16%)	253 (24.3%)	
Male	353 (33.9%)	268 (25.7%)	
Race, n (%)			0.192
Asian	12 (1.4%)	5 (0.6%)	
Black or African American	41 (4.8%)	44 (5.1%)	
White	367 (42.6%)	392 (45.5%)	
Age, n (%)			0.536
<= 65	230 (22.7%)	218 (21.5%)	
> 65	279 (27.5%)	286 (28.2%)	
Residual tumor, n (%)			0.397
R0	394 (49.9%)	362 (45.9%)	
R1	12 (1.5%)	13 (1.6%)	
R2	6 (0.8%)	2 (0.3%)	
Anatomic neoplasm subdivision, n (%)			0.049
Bronchial	7 (0.7%)	3 (0.3%)	
Left	224 (22.2%)	197 (19.6%)	
Right	268 (26.6%)	308 (30.6%)	
Location, n (%)			0.015
Central Lung	123 (28.6%)	87 (20.2%)	
Peripheral Lung	103 (24%)	117 (27.2%)	
Number pack years smoked, n (%)			0.008
< 40	144 (18.1%)	178 (22.4%)	
>= 40	256 (32.2%)	216 (27.2%)	
Neoplasm type, n (%)			< 0.001
Adenocarcinoma	164 (15.8%)	375 (36%)	
Squamous Cell Carcinoma	356 (34.2%)	146 (14%)	
OS event, n (%)			0.010
Alive	296 (28.4%)	337 (32.4%)	
Dead	224 (21.5%)	184 (17.7%)	

### Decreased *FAM20A* is not correlated with LUSC prognosis

**Figure 4:**
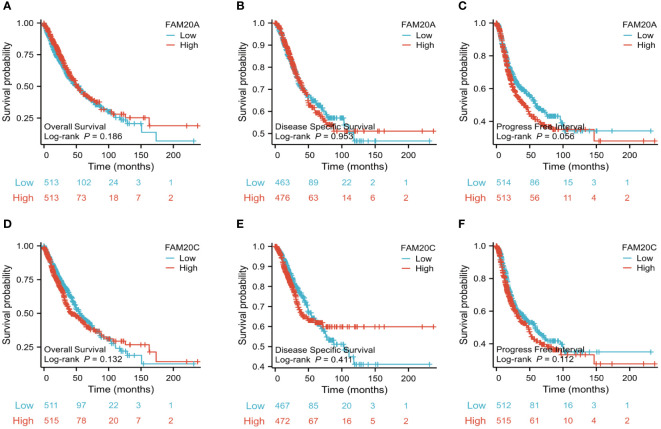
Association of FAM20A and FAM20C levels with Clinical Pathological Traits in LUSC. **(A)** Overall survival, **(B)** Disease specific survival and **(C)** Progress Free interval of *FAM20A* mRNA levels. **(D)** Overall survival, **(E)** Disease specific survival and **(F)** Progress Free interval of *FAM20C* mRNA levels. Significant levels indicated as *P<0.05, ** *P*<0.01, *** *P*<0.001. LUSC, Lung squamous cell carcinoma; Fam20A, Family with Squance Similarity 20, member A; Fam20C, Family with Squance Similarity 20, member C.

**Figure 5:**
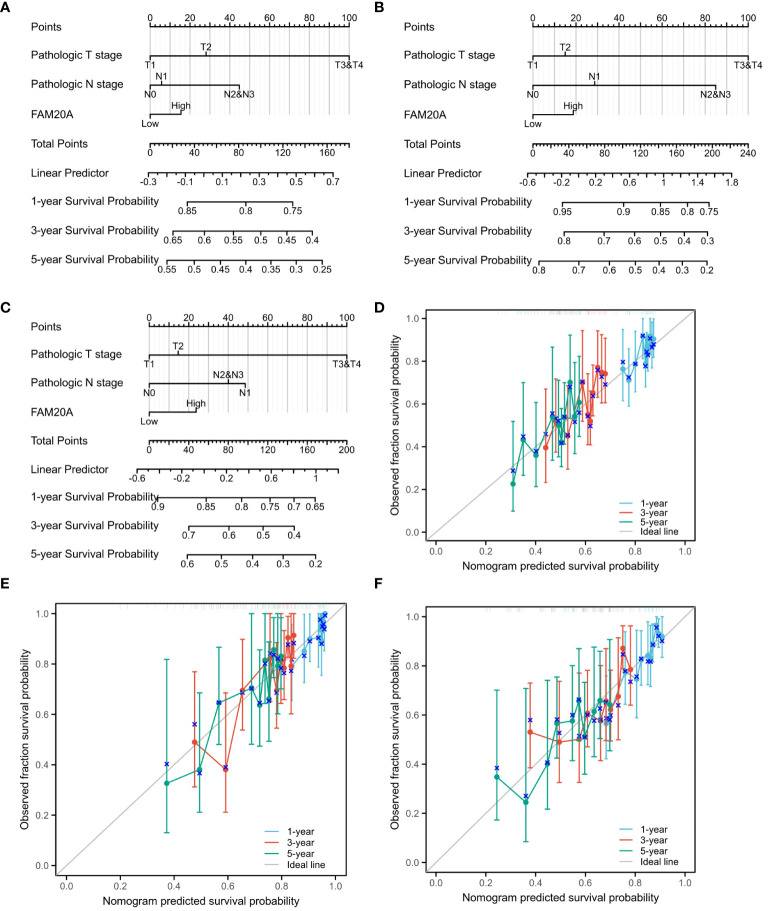
Nomogram Predictions and Calibration for LUSC Patients. A nomogram predicting the first-year, three-year, and five-year outcomes for **(A)** OS, **(B)** DSS, **(C)** and PFI. Calibration plots **(D-F)** for OS, DSS, and PFI validate the predictive accuracy of the nomogram. LUSC, Lung squamous cell carcinoma; FAM20A, Family with Sequence Similarity 20, Member A; OS, Overall survival; DSS, Disease-specific survival; PFI, Progression-free interval.

Given the importance of prognosis in LUSC patient outcomes, we investigated the relationship between *FAM20A*/*FAM20C* expression levels and LUSC prognosis using the CGGA dataset. In a cohort of 1,026 paired patients, decreased *FAM20A* expression did not correlate with overall survival (OS, **Figure 4A**), disease-specific survival (DSS, **Figure 4B**), or progression-free survival (PFS, **Figure 4C**) (*P*>0.05). Additionally, these patients showed no significant prognostic correlation in OS (**Figure 4D**), DSS (**Figure 4E**), and PFS (**Figure 4F**) with *FAM20C* expression levels (*P*>0.05). We further developed a nomogram to predict the prognosis of LUSC patients, encompassing OS (**Figure 4A**), DSS (**Figure 4B**), and PFI (**Figure 4C**), and validated it using calibration curves. The nomogram’s calibration curves for 1-, 3-, and 5-year clinical outcomes of OS (**Figure 4D**), DSS (**Figure 4E**), and PFI (**Figure 4F**) showed alignment with the 45-degree line, indicating a match between predicted and observed values. Subsequently, we performed nomogram prediction and calibration of LUSC patients with the first-year, three-year, and five-year outcomes for OS (**Figure 5A**), DSS (**Figure 5B**), and PFI (**Figure 5C**). We calibrate these plots (**Figure 5D-F**) to validate the predictive accuracy of the nomogram. These results suggest no correlation between *FAM20A* expression levels and LUSC outcomes.

### Correlation between *FAM20A* and other oncogenes

**Figure 6:**
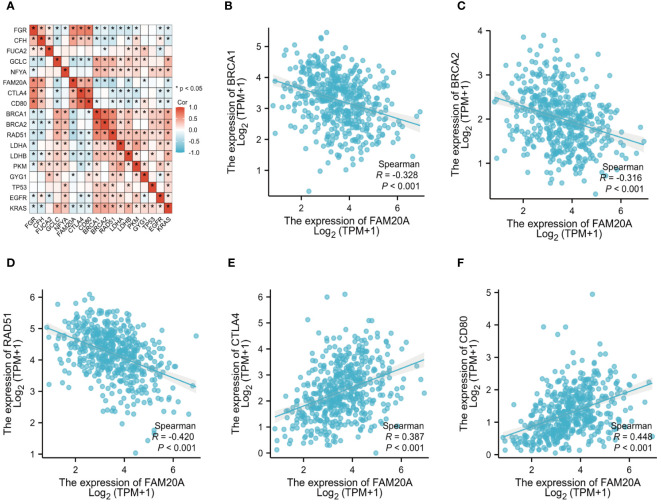
FAM20A and Its Correlation with Oncogenes. **(A)** Analysis of *FAM20A*’s correlation with various oncogenes. The correlation of *FAM20A* with **(B)** *BRCA1*, **(C)** *BRCA2*, **(D)** *RAD51*, **(E)** *CTLA4*, and **(F)** *CD80*. FAM20A, Family with Sequence Similarity 20, Member A; FGR, Gardner-Rasheed feline sarcoma viral (v-fgr) oncogene homolog; CFH, Complement factor H; FUCA2, Alpha-L-fucosidase 2; GCLC, Glutamate-cysteine ligase catalytic subunit; NFYA, Nuclear transcription factor Y subunit alpha; CTLA4, Cytotoxic T-lymphocyte associated protein 4; BRCA1, Breast Cancer 1 DNA repair associated; BRCA2, Breast Cancer 2 DNA repair associated; RAD51, Radiation sensitive 51 recombinase; LDHA, Lactate dehydrogenase A; LDHB, Lactate dehydrogenase B; PKM, Pyruvate kinase M1/2; GYG1, Glycogenin 1; TP53, Tumor protein p53; EGFR, Epidermal growth factor receptor; KRAS, Kirsten Rat Sarcoma proto-oncogene, GTPase.

To delve deeper into *FAM20A*’s role in the tumor microenvironment, metabolism, and DNA repair, we examined its correlation with various oncogenes. **Table 3** details the abbreviations and full names of these oncogenes and DNA-repair-related genes. **Figures 6** and **Supplementary Figure S1** illustrate that in the TCGA LUSC dataset, *FAM20A* negatively correlates with genes such as *Breast Cancer 1 DNA Repair Associated (BRCA1), Breast Cancer 2 DNA Repair Associated (BRCA2), Radiation sensitive 51 Recombinase (RAD51), Kirsten Rat Sarcoma (KRAS), B-Raf Proto-Oncogene (BRAF), B-Cell Lymphoma 2 (BCL2), Beclin 1 (BECN1), Nuclear Factor, Erythroid 2 Like 2 (NFE2L2), and Ferredoxin 1 (FDX1).* In contrast, a positive correlation exists between *FAM20A* expression and the expression of *Cytotoxic T-Lymphocyte Associated Protein 4 (CTLA4)*, *CD80*, *Receptor Interacting Serine/Threonine Kinase 3 (RIPK3)*, *Receptor Interacting Serine/Threonine Kinase 1 (RIPK1)*, *Gasdermin A (GSDMA)*, *ROS Proto-Oncogene 1 (ROS1)*, *Acyl-CoA Synthetase Long Chain Family Member 4 (ACSL4)*, and *Hypoxia Inducible Factor 1 Subunit Alpha (HIF-1α)*. However, no significant correlation was observed between *FAM20A* expression and the expression of *Tumor Protein 53 (TP53), Epidermal Growth Factor Receptor (EGFR), Anaplastic Lymphoma Kinase (ALK), MET Proto-Oncogene (MET), BCL2 Associated X (BAX)*, and *Sirtuin 1* (*SIRT1)*.

**Table 3:** Abbreviations and details of the oncogenes and DNA-repair-related genes.

Abbreviation	Detail
Acyl-CoA synthetase long-chain family	ACSL4
Anaplastic Lymphoma kinase	ALK
BCL2-Associated X	BAX
B-cell lymphoma-2	BCL2
Myosin-like BCL2-interacting protein	BECN1
B-Raf proto-oncogene, serine/threonine kinase	BRAF
Breast cancer susceptibility gene 1	BRCA1
Breast cancer susceptibility gene 2	BRCA2
Cytotoxic T-Lymphocyte Associated Antigen 4	CTLA4
Epidermal Growth Factor-like repeat A	EGFA
Ferredoxin 1	FDX1
Gasdermin A	GSDMA
Hypoxia-inducible factor-1α	HIF-1α
Kirsten rats sarcoma viral oncogene homolog	KRAS
Mesenchymal to epithelial transition factor	MET
NFE2 Like BZIP Transcription Factor 2	NFE2L2
RAD51 Recombinase	RAD51
Receptor-interacting serine/threonine-protein kinase 1	RIPK1
Receptor-interacting serine/threonine-protein kinase 3	RIPK3
ROS proto-oncogene 1, receptor tyrosine kinase	ROS1
Silent Information Regulator 1	SIRT1
Tumor protein p53	TP53

### *FAM20A* correlation with cancer immunology

**Figure 7:**
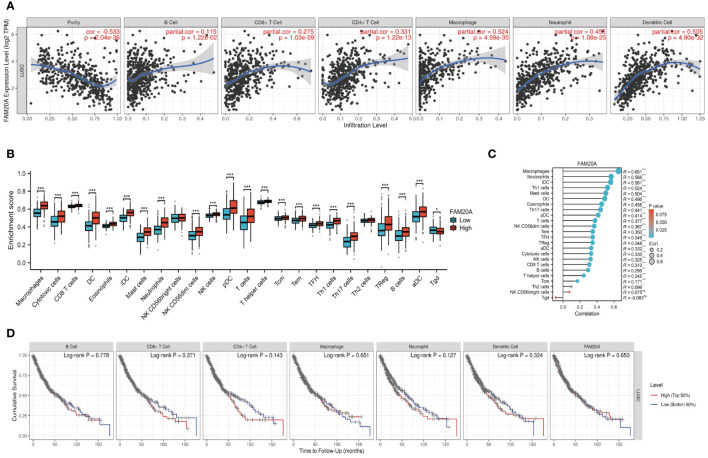
FAM20A and Immune Infiltration in LUSC. **(A)** Analysis of the relationship between *FAM20A* expression and immune infiltration levels in LUSC, using TIMER databases. **(B)** Correlation between *FAM20A* expression and 24 immune cell types in LUSC tissues, as per TCGA data. **(C)** Correlation analysis in LUSC tissues from GTEx data. **(D)** Cumulative survival analysis of LUSC patients considering immune cell presence and *FAM20A* levels. Significance levels indicated as *P<0.05, ***P<0.001. LUSC, Lung squamous cell carcinoma; FAM20A, Family with Sequence Similarity 20, Member A; TIMER, Tumor Immune Estimation Resource; TCGA, The Cancer Genome Atlas; DC, Dendritic cells; iDC, Immature dendritic cells; pDC, Plasmacytoid dendritic cells; Tcm, Central memory T cells; Tem, Effector memory T cells; TFH, Follicular helper T cells; aDC, Activated dendritic cells; Tgd, Gamma delta T cells; NK cells, Natural killer cells; Treg, Regulatory T cells.

Building on previous studies that link the prognosis of various cancers to different immune cell types (22), we examined the association between *FAM20A* expression and immune cells in LUSC patients. We discovered that *FAM20A* expression inversely correlates with tumor purity in LUSC, and positively correlates with the presence of B cells, CD8^+^ T cells, CD4^+^ T cells, macrophages, neutrophils, and dendritic cells (**Figure 7A**). To further dissect these correlations, we categorized *FAM20A* expression levels as low or high and then correlated these levels with different immune cell subtypes. As depicted in **Figure 7B** and **Supplementary Figure S2**, high *FAM20A* expression was significantly upregulated in 21 immune cell types, including macrophages, cytotoxic cells, CD8^+^ T cells, dendritic cells (DC), eosinophils, immature DC (iDC), mast cells, neutrophils, NK CD56 dim cells, NK cells, plasmacytoid DC (pDC), T cells, T helper cells, effector memory T cells (Tem), central memory T cells (Tcm), Tfh, Th1 cells, Th17 cells, Th2 cells, Tregs, B cells, and activated DC (aDC) (all *P*<0.05). In contrast, *FAM20A* expression showed a negative correlation with γδ T cells (**Figures 7B, C**, *P*<0.05) and no significant correlation with NK CD56bright cells (*P*>0.05). Although *FAM20A* expression levels are positively associated with most immune cells, the degree of immune cell infiltration did not correlate with survival outcomes (**Figure 7D**, *P*>0.05).

### Methylation and gene set enrichment of *FAM20A*

**Figure 8:**
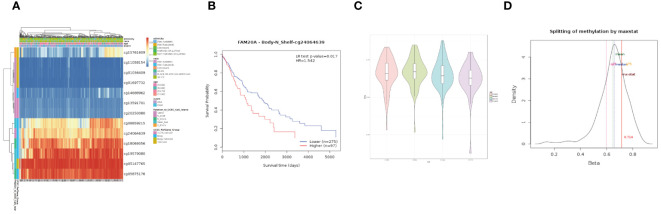
DNA Methylation of *FAM20A* in LUSC. **(A)** A heatmap depicting *FAM20A* DNA methylation expression levels in LUSC, using the MethSurv platform. **(B)** Prognostic significance of *FAM20A* cg24064639 methylation in LUSC. **(C)** Violin plots comparing methylation levels across different age groups. **(D)** Methylation level density distribution. LUSC, Lung squamous cell carcinoma; FAM20A, Family with Sequence Similarity 20, Member A.

**Figure 9:**
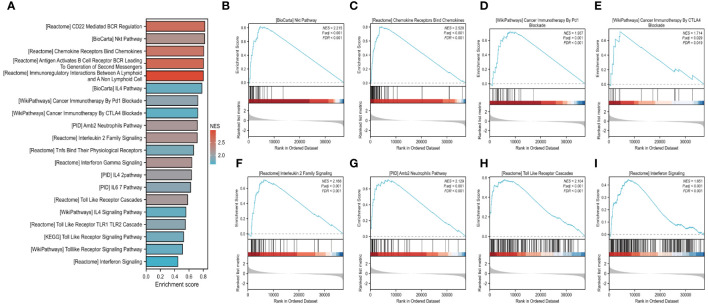
GSEA analysis of LUSC in *FAM20A* expression groups. Gene set enrichment analysis (GSEA) conducted on LUSC transcriptomic data from the TCGA database **(A)**. Eight of these pathways, pertinent to the tumor microenvironment, including the Nkt Pathway **(B)**, Chemokine Receptors Bind Chemokines **(C)**, Cancer Immunotherapy by PD1 Blockade **(D)**, Cancer Immunotherapy by CTLA-4 Blockade **(E)**, Interleukin 2 Family Signaling **(F)**, Amb2 Neutrophils Pathway **(G)**, Toll-Like Receptor Cascades **(H)**, Interferon Signaling **(I)**.

We further investigated the link between *FAM20A* methylation and LUSC prognosis. As illustrated in **Figure 8A**, the MetSurv tool identified 13 methylated CpG islands on the *FAM20A* chromosome, including cg08859215, cg24064639, cg18069356, cg19579080, cg05147765, and cg05875176. Notably, cg24064639 methylation was associated with a poorer prognosis (**Figure 8B**). Additionally, no significant differences were found in the methylation levels among LUSC patients of varying ages (**Figures 8C, D**). Gene set enrichment analysis (GSEA) conducted on LUSC transcriptomic data from the TCGA database revealed 20 pathways related to immune therapy and regulation (**Figure 9A**). Eight of these pathways, pertinent to the tumor microenvironment, including the Nkt Pathway, Chemokine Receptors Bind Chemokines, Interleukin 2 Family Signaling, Toll-Like Receptor Cascades, Interferon Signaling, Cancer Immunotherapy by PD1 Blockade, Cancer Immunotherapy by CTLA4 Blockade, and Amb2 Neutrophils Pathway, exhibited significant enrichment in the high *FAM20A* expression group, as indicated by NES, FDR, and p-values (**Figures 9B-I**).

## Discussion

Lung cancer, and specifically lung squamous cell carcinoma (LUSC), remains a leading cause of cancer mortality globally. The ineffectiveness of current treatments, including surgical resection, chemotherapy, and immunotherapy, underscores the urgency of early intervention strategies. This study aims to identify novel diagnostic biomarkers for LUSC.

By single-cell sequencing analysis, we observed that *FAM20A* is specifically expressed in alveolar cells. Whereas, compared with the healthy controls, the transcription levels of *FAM20A* are greatly reduced in tumor cells. Then, utilizing multiple public databases, we identified *FAM20A* as a potential tumor-suppressive gene at all LUSC stages. Its expression positively correlates with immune cells, suggesting that decreased *FAM20A* levels in LUSC patients may contribute to tumor cell immune evasion. Moreover, *FAM20A* downregulation is linked to enhanced DNA repair signaling pathways, potentially leading to radiotherapy resistance. Methylation and gene set enrichment analyses also indicated that *FAM20A* hypermethylation is associated with poor LUSC prognosis, and several immune-related signaling pathways are regulated by *FAM20A* expression. Collectively, our findings position *FAM20A* as a novel tumor suppressor in LUSC, potentially influencing tumor immune interactions and serving as a prognostic biomarker. Currently, the majority of LUSC patients are diagnosed at advanced stages, underscoring the need for early detection strategies. Analysis of FAM20A may enable early diagnosis, facilitating timely intervention to effectively manage LUSC progression. This study initially investigated the expression levels and clinical relevance of FAM20A in LUSC, demonstrating that FAM20A made it feasible to distinguish between LUSC and non-LUSC specimens and the novel and potential biomarker for the prediction and treatment of LUSC. Although our study clarified that FAM20A has an inhibitory effect on LUSC, the mechanism of FAM20A’s decline in LUSC is not well understood.

Previous studies have highlighted *FAM20A*’s pivotal role in tissue development and mineralization (5, 23). As a pseudoenzyme with limited enzymatic function, most research on FAM20A has focused on its interaction with FAM20C and regulation of FAM20C localization (24). FAM20A activates FAM20C by forming a heterodimer or tetrameric complex with FAM20C (5). The formation of heterodimers can significantly promote the kinase activity of FAM20C, and the two heterodimers can further combine to form heterotetrameric complexes (9, 10). Heterodimer formation is sufficient to allosterically activate FAM20C activity *in vitro* and in cells, but the unique contribution of heterotetramers remains unknown (9). The FAM20A-FAM20C interaction occurs in a FAM20C kinase activity-independent manner (24), enhancing its phosphorylation of secreted proteins that are essential for biomineralization (25). Additionally, FAM20A controls the extracellular localization of FAM20C and is required for FAM20C secretion (24). The phenotypic overlap observed in *Fam20a* and *Fam20c* knockout mice and patients with mutations in *FAM20A* and *FAM20C*, including conditions such as amelogenesis imperfecta, gingival fibromatosis, ectopic calcification, and periodontal disease, indicates the close correlation between FAM20A and FAM20C in structural, functional, and developmental aspects (24). Our study is the first to explore *FAM20A* in LUSC. Unexpectedly, despite identifying *FAM20A* as a tumor suppressor in LUSC, its expression did not correlate with patient prognosis. We analyzed overall survival, disease-free survival, and progression-free interval, finding no significant association with *FAM20A* levels. This might be attributed to the treatments received by these patients, predominantly surgery and chemotherapy. Our immune cell analysis revealed a positive correlation between *FAM20A* and immune cells, suggesting that decreased *FAM20A* in LUSC might lead to diminished immune responses. Tumor-infiltrating lymphocytes (TILs) are crucial in the immune response against tumors (26). In LUSC, a lower percentage of TILs in conjunction with reduced *FAM20A* expression implies a potential role in tumor immune evasion. Our data revealed a correlation between *FAM20A* levels and increased neutrophil and dendritic cell abundance. Neutrophils exert significant anti-tumor effects (27, 28), and our findings suggest that reduced *FAM20A* expression in all LUSC stages may impair immune responses to cancer cells. Similarly, dendritic cells, known for their anti-tumor responses through antigen presentation and T cell activation, also showed a positive correlation with *FAM20A*, whether plasmacytoid or activated. Regulatory T cells (Tregs), known for their immunosuppressive functions, were also examined (29). GSEA enrichment analysis identified immune-related signaling pathways predominantly enriched in the high *FAM20A* expression group, indicating complex regulatory mechanisms of *FAM20A* on immune responses in LUSC. Our research indicates a positive correlation between FAM20A and immune cells, implying its pivotal role in modulating the tumor immune microenvironment (TIME) in LUSC. This offers novel insights into enhancing anti-tumor immune responses through targeted modulation of FAM20A. While immune checkpoints serve as indicators for immunotherapy benefits, they do not encompass the entire complexity of the tumor immune microenvironment. Therefore, comprehensive biomarkers evaluating the TIME beyond immune checkpoints and tumor mutations are imperative. FAM20A holds promise in fulfilling this role in LUSC treatment, enhancing therapeutic efficacy, and offering more precise treatment strategies for LUSC patients.

In response to various intracellular and extracellular stress stimuli, organisms can initiate autophagy, cuproptosis, pyroptosis, ferroptosis, and necrotic apoptosis to inhibit cancer cell proliferation, while also playing a synergistic role in anti-tumor immune responses (30). Activation of receptor-interacting protein kinases (RIPK1, RIPK3) promotes upregulation of chemokines, facilitating the infiltration of CD8 T cells and thereby enhancing anti-tumor immunity (31). Our study observed a negative correlation between *FAM20A* and apoptotic molecules like BCL2, autophagy-related BECN1, and cuproptosis-related FDX1. Conversely, *FAM20A* positively correlated with necrosis-related RIPK1, RIPK3, and pyroptosis-related GSDMA. NFE2L2 and ACSL4, both involved in ferroptosis, showed a negative and positive correlation with *FAM20A*, respectively.

Homologous recombination (HR) is a crucial DNA repair mechanism for double-strand breaks (DSB) (32). BRCA1 and BRCA2 are crucial for HR and DSB repair. In addition, RAD51 filaments are critical intermediates in HR. BRCA1, BRCA2, and RAD51 are all key participants in DNA damage repair and show a promoting role in tumor metastasis (33–35). Our experiments have demonstrated a certain relationship between *FAM20A* and BRCA1, BRCA2, and RAD1 in LUSC. Our findings reveal the association of FAM20A with DNA repair signaling pathways and its impact on radiotherapy resistance in LUSC patients. This provides novel insights into the mechanisms underlying LUSC treatment responses, suggesting FAM20A as a potential predictive biomarker for radiotherapy efficacy, addressing challenges of targeted therapy resistance in LUSC.

KRAS transmits signals from the cell surface to the nucleus, influencing tumor cell proliferation (36). Oncogenic ROS1 rearrangement is a therapeutic target in lung cancer (37). HIF-1α, a biomarker of tumor hypoxia, is upregulated in hypoxic conditions and is associated with tumor progression and metastasis (38). Our experimental findings also demonstrated the association between *FAM20A* and these molecules in LUSC. CTLA-4 is a key protein associated with tumor immune evasion (39). CTLA-4 inhibitors, such as ipilimumab, have demonstrated favorable efficacy in the treatment of melanoma, colorectal cancer (CRC), and hepatocellular carcinoma (HCC) (40). This study analyzed the relationship between CTLA-4 and *FAM20A* and found a positive correlation in their expression in LUSC. CTLA-4 interacts with CD80, thereby limiting T-cell activation and leading to immune dysfunction (41). Interestingly, the level of CD80 and *FAM20A* was also favorably correlated.

DNA methylation is a ubiquitous epigenetic mechanism that plays a crucial role in tumor development (42). Methylation of CpG sites in the promoter region is believed to influence the process of DNA transcription by silencing the transcription factors (43), and without altering the DNA sequence. The methylation of AKAP13, nuclear receptor binding SET domain protein 3 (NSD3), and FUT7 are connected with the diagnosis of LUSC patients (44, 45). Our study indicated that hypermethylation of cg24064639 is associated with poorer prognosis, suggesting that epigenetic changes might impact LUSC progression by inhibiting *FAM20A* transcription.

In summary, our study proposes an association between *FAM20A* and tumor immunity, positioning it as a potential immune therapy target in LUSC. While *FAM20A* may play a role in LUSC prognostic diagnosis, our experiments, primarily based on database data, have limitations. Further research is needed to unravel the mechanisms by which *FAM20A* influences LUSC progression.

## Data availability statement

The datasets presented in this study can be found in online repositories. The names of the repository/repositories and accession number(s) can be found in the article/**Supplementary Material**.

## Ethics statement

The human samples used in this study conformed to national and institutional ethical guidelines and were approved by the Clinical Research Ethics Committee of Sichuan Provincial People’s Hospital. The studies were conducted in accordance with the local legislation and institutional requirements. Written informed consent for participation in this study was provided by the participants’ legal guardians/next of kin.

## Author contributions

YZ: Data curation, Writing – original draft. QS: Formal analysis, Writing – original draft. YL: Data curation, Writing – original draft. XY: Software, Writing – original draft. HW: Data curation, Methodology, Resources, Writing – original draft. SS: Investigation, Methodology, Resources, Software, Validation, Writing – review & editing. ZY: Funding acquisition, Writing – review & editing. YW: Conceptualization, Funding acquisition, Supervision, Validation, Visualization, Writing – review & editing. YF: Funding acquisition, Project administration, Validation, Writing – review & editing.

## Supplementary material

Supplementary Figure 1 Spearman correlation coefficients of *FAM20A* and oncogenes, cell death and hypoxia. **(A)** Correlation of *FAM20A* with *TP53*, *KRAS* (*P*<0.001), *EGFR*, *ALK*, *MET* and *BRAF* (*P*<0.001). **(B)** Correlation of *FAM20A* with apoptosis genes (*BCL2* and *BAX*). (*P*<0.05 for *BCL2*) **(C)** Correlation of *FAM20A* with autophage (*BECN1*) (*P*<0.01); **(D)** Correlation of *FAM20A* with necroptosis (RIPK1 and RIPK3) (*P*<0.001); **(E)** Correlation of *FAM20A* with pyroptosis (*GASDMA*) (*P*<0.001); **(F)** Correlation of *FAM20A* with ferroptosis (*ROS1* and ACSL4) (*P*<0.001); **(G)** Correlation of *FAM20A* with cuproptosis (*FDX1*) (*P*<0.05); **(H)** Correlation of *FAM20A* with *SIRT1* (*P*>0.05); **(I)** Correlation of *FAM20A* with hypoxia (*HIF1A*) (*P*<0.001). TP53, Tumor protein p53; KRAS, KRAS proto-oncogene, GTPase; EGFR, Epidermal growth factor receptor; ALK, Anaplastic lymphoma kinase; BCL2, B-cell CLL/lymphoma 2; BAX, BCL2 associated X, apoptosis regulator; BECN1, Beclin 1; RIPK3, Receptor interacting serine/threonine kinase 3; RIPK1, Receptor interacting serine/threonine kinase 1; GSDMA, Gasdermin A; ROS1, ROS proto-oncogene 1, receptor tyrosine kinase; ACSL4, Acyl-CoA synthetase long chain family member 4; NFE2L2, Nuclear factor, erythroid 2 like 2; FDX1, Ferredoxin 1; SIRT1, Sirtuin 1; HIF1A, Hypoxia inducible factor 1 subunit alpha.

Supplementary Figure 2 Spearman correlation coefficients of *FAM20A* and immune cells. The immune cells include macrophages (**(A)** *P*<0.001), neutrophiles (**(B)** *P*<0.001), mast cells (**(C)** *P*<0.001), eosinophils (**(D)** *P*<0.001), B cells (**(E)** *P*<0.001), DC (**(F)** *P*<0.001), iDC (G, *P*<0.001), pDC (**(H)** *P*<0.001), aDC (**(I)** *P*<0.001), NK (**(J)** *P*<0.001), CD56dim cells (**(K)** *P*<0.001), CD56bright cells (**(L)** *P*=0.092), T cells (**(M)** *P*<0.001), cytotoxic cells (**(N)** *P*<0.001), CD8 T cells (**(O)** *P*<0.001), Tem (**(P)** *P*<0.001), Tcm (**(Q)** *P*<0.001), Tfh (**(R)** *P*<0.001), T helper cells (**(S)** *P*<0.001), Th1 cells (**(T)** *P*<0.001), Th2 cells **(U)** *P*<0.05), Th17 cells **(V)**, *P*<0.001, Tgd (**(W)** *P*=0.062), Treg (**(X)** *P*<0.001). DC, Dendritic cells; iDC, Immature dendritic cells; pDC, Plasmacytoid dendritic cells; aDC, Activated dendritic cells; NK cells, Natural killer cells; Tcm, Central memory T cells; Tem, Effector memory T cells; TFH, Follicular helper T cells; Treg, Regulatory T cells.
