# Supplementary figures and images for: FAM20A: a potential diagnostic biomarker for lung squamous cell carcinoma
Source: Front Immunol. 2024 Jun 25;15:1424197. doi: 10.3389/fimmu.2024.1424197 (PMC11231076; doi:10.3389/fimmu.2024.1424197)

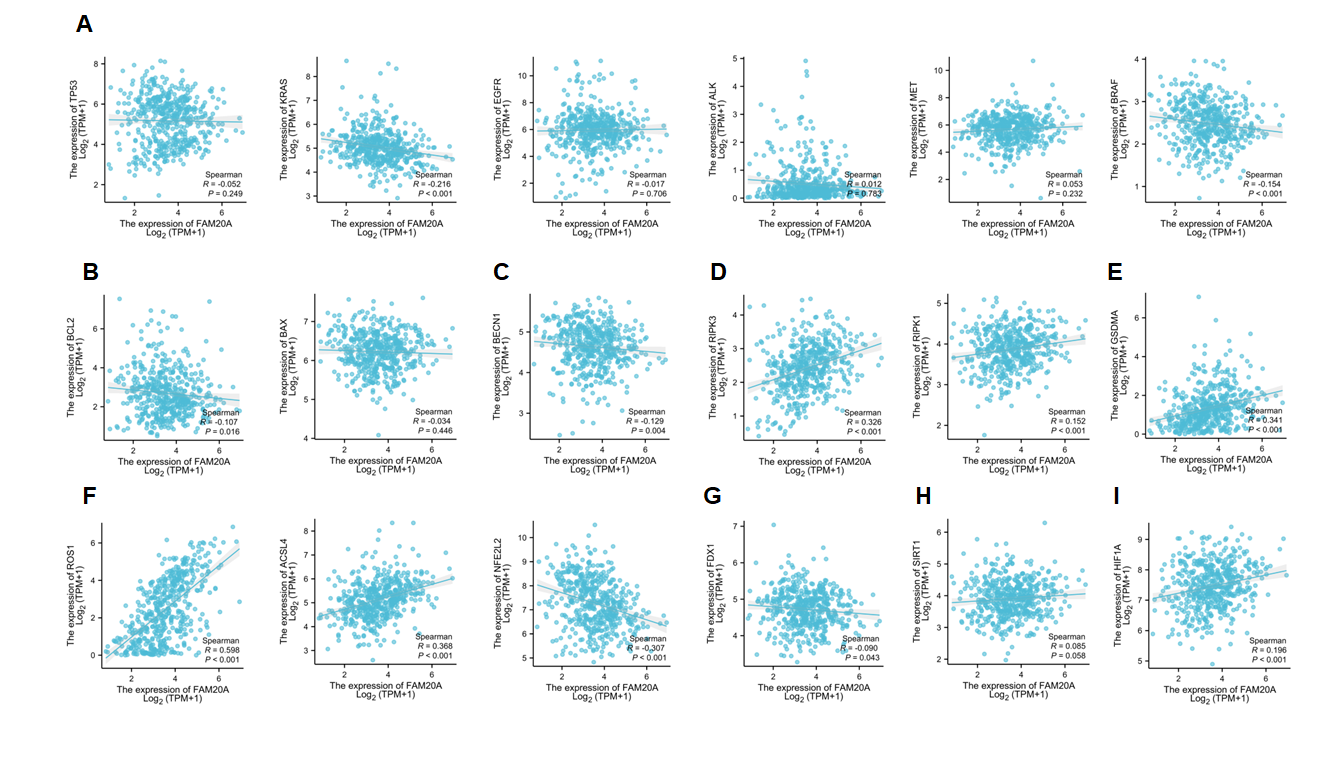

Supplement: Supplementary Figure 1 — Spearman correlation coefficients of FAM20A and oncogenes, cell death and hypoxia. (A) Correlation of FAM20A with TP53, KRAS (P<0.001), EGFR, ALK, MET and BRAF (P<0.001). (B) Correlation of FAM20A with apoptosis genes (BCL2 and BAX). (P<0.05 for BCL2) (C) Correlation of FAM20A with autophage (BECN1) (P<0.01); (D) Correlation of FAM20A with necroptosis (RIPK1 and RIPK3) (P<0.001); (E) Correlation of FAM20A with pyroptosis (GASDMA) (P<0.001); (F) Correlation of FAM20A with ferroptosis (ROS1 and ACSL4) (P<0.001); (G) Correlation of FAM20A with cuproptosis (FDX1) (P<0.05); (H) Correlation of FAM20A with SIRT1 (P>0.05); (I) Correlation of FAM20A with hypoxia (HIF1A) (P<0.001). TP53, Tumor protein p53; KRAS, KRAS proto-oncogene, GTPase; EGFR, Epidermal growth factor receptor; ALK, Anaplastic lymphoma kinase; BCL2, B-cell CLL/lymphoma 2; BAX, BCL2 associated X, apoptosis regulator; BECN1, Beclin 1; RIPK3, Receptor interacting serine/threonine kinase 3; RIPK1, Receptor interacting serine/threonine kinase 1; GSDMA, Gasdermin A; ROS1, ROS proto-oncogene 1, receptor tyrosine kinase; ACSL4, Acyl-CoA synthetase long chain family member 4; NFE2L2, Nuclear factor, erythroid 2 like 2; FDX1, Ferredoxin 1; SIRT1, Sirtuin 1; HIF1A, Hypoxia inducible factor 1 subunit alpha. [file Image_1.tif]

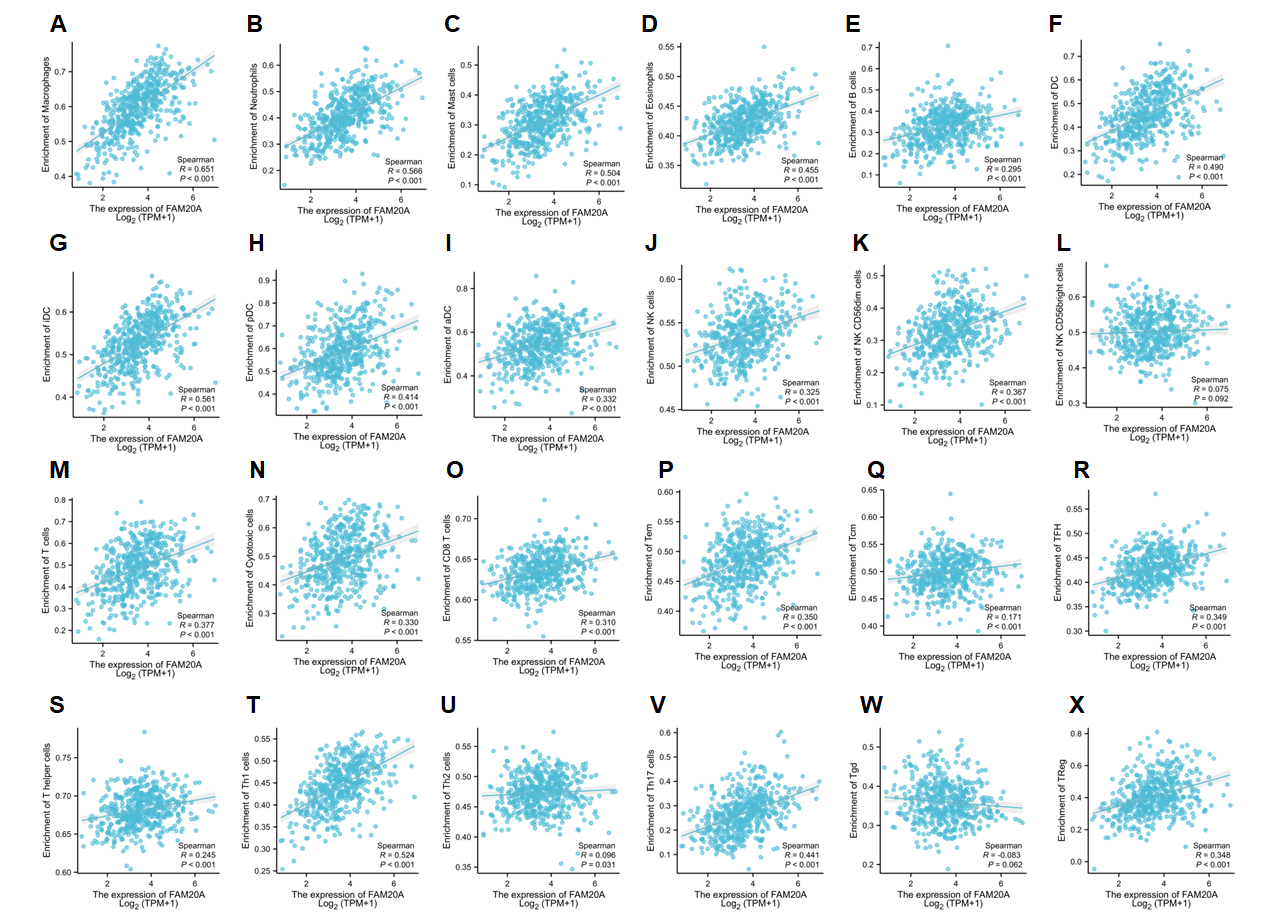

Supplement: Supplementary Figure 2 — Spearman correlation coefficients of FAM20A and immune cells. The immune cells include macrophages ((A) P<0.001), neutrophiles ((B) P<0.001), mast cells ((C) P<0.001), eosinophils ((D) P<0.001), B cells ((E) P<0.001), DC ((F) P<0.001), iDC (G, P<0.001), pDC ((H) P<0.001), aDC ((I) P<0.001), NK ((J) P<0.001), CD56dim cells ((K) P<0.001), CD56bright cells ((L) P=0.092), T cells ((M) P<0.001), cytotoxic cells ((N) P<0.001), CD8 T cells ((O) P<0.001), Tem ((P) P<0.001), Tcm ((Q) P<0.001), Tfh ((R) P<0.001), T helper cells ((S) P<0.001), Th1 cells ((T) P<0.001), Th2 cells (U) P<0.05), Th17 cells (V), P<0.001, Tgd ((W) P=0.062), Treg ((X) P<0.001). DC, Dendritic cells; iDC, Immature dendritic cells; pDC, Plasmacytoid dendritic cells; aDC, Activated dendritic cells; NK cells, Natural killer cells; Tcm, Central memory T cells; Tem, Effector memory T cells; TFH, Follicular helper T cells; Treg, Regulatory T cells. [file Image_2.tif]
